# Sequencing, identification and mapping of primed L1 elements (SIMPLE) reveals significant variation in full length L1 elements between individuals

**DOI:** 10.1186/s12864-015-1374-y

**Published:** 2015-03-21

**Authors:** Vincent A Streva, Vallmer E Jordan, Sara Linker, Dale J Hedges, Mark A Batzer, Prescott L Deininger

**Affiliations:** Tulane Cancer Center and Department of Epidemiology, Tulane University, New Orleans, LA USA; Department of Biology, Louisiana State University, Baton Rouge, LA USA; Department of Human Genetics, University of Miami Miller School of Medicine, Miami, FL USA; Department of Internal Medicine, The Ohio State University, Columbus, OH USA; Present Address: Division of Infectious Diseases, Boston Children’s Hospital and Harvard Medical School, Boston, MA USA

**Keywords:** Retrotransposon, High-throughput sequencing, LINE1, Polymorphism

## Abstract

**Background:**

There are over a half a million copies of L1 retroelements in the human genome which are responsible for as much as 0.5% of new human genetic diseases. Most new L1 inserts arise from young source elements that are polymorphic in the human genome. Highly active polymorphic “hot” L1 source elements have been shown to be capable of extremely high levels of mobilization and result in numerous instances of disease. Additionally, hot polymorphic L1s have been described to be highly active within numerous cancer genomes. These hot L1s result in mutagenesis by insertion of new L1 copies elsewhere in the genome, but also have been shown to generate additional full length L1 insertions which are also hot and able to further retrotranspose. Through this mechanism, hot L1s may amplify within a tumor and result in a continued cycle of mutagenesis.

**Results and conclusions:**

We have developed a method to detect full-length, polymorphic L1 elements using a targeted next generation sequencing approach, Sequencing Identification and Mapping of Primed L1 Elements (SIMPLE). SIMPLE has 94% sensitivity and detects nearly all full-length L1 elements in a genome. SIMPLE will allow researchers to identify hot mutagenic full-length L1s as potential drivers of genome instability. Using SIMPLE we find that the typical individual has approximately 100 non-reference, polymorphic L1 elements in their genome. These elements are at relatively low population frequencies relative to previously identified polymorphic L1 elements and demonstrate the tremendous diversity in potentially active L1 elements in the human population.

**Electronic supplementary material:**

The online version of this article (doi:10.1186/s12864-015-1374-y) contains supplementary material, which is available to authorized users.

## Background

Mobile elements have been extremely successful at colonizing human genomes. As much as two thirds of human genomic content is either made up of mobile elements or mobile element relics [1,2]. The two most significant classes of human mobile elements are long interspersed elements (LINEs) and short interspersed elements (SINEs), and together, these elements account for at least 38% of genomic content [2,3]. In humans, the most significant members of the LINE and SINE families are L1 and Alu, respectively.

L1 elements have been amplifying in mammalian genomes since before the divergence of placental and marsupial mammals 170 million years ago [4]. Since becoming established in primate genomes (~40 million years ago), there has been a linear evolution of L1 subfamilies, with each newly active L1 subfamily replacing the one that came before [5,6]. Today, the predominant active L1Hs family of L1 retrotransposons is responsible for the majority of known L1 retrotransposition events [4,7-10]. L1 promoters and other functional sequences vital to L1 retrotransposition accumulate disruptive mutations as a function of time. Hence, it is not surprising that studies have shown the elements most likely to be active are those that have most recently inserted and therefore may not have become fixed in the population [11-13]. These polymorphic L1 elements are often capable of significant levels of L1 retrotransposition [11,14].

Active full length L1 is a ~6 kb long element that codes for two open reading frames (ORFs): ORF1 which encodes a protein with nucleic acid chaperone and RNA binding properties, and ORF2 which encodes a protein with endonuclease (EN) and reverse transcriptase (RT) activities [15,16]. L1 mobilizes through a process called retrotransposition [17,18]. Briefly, full length, intact L1 loci are transcribed by RNA polymerase II, to generate a bicistronic L1 mRNA encoding two proteins, ORF1p and ORF2p [19]. The L1 mRNA and L1 proteins form a ribonucleoprotein (L1 RNP) which enters the nucleus where the ORF2p-encoded endonuclease (EN) and reverse transcriptase (RT) create a cDNA copy of the L1 mRNA in a new genomic location through a process called target primed reverse transcription (TPRT) [17,18]. Through retrotransposition, L1 has been able to amplify itself to its current copy number of ~500,000 copies per genome. However, the majority of these L1 elements are non-functional relics due to severe truncation at their 5’ ends caused by aborted TPRT events and accumulation of deleterious mutations either over time or as a result of unfaithful RT activity [2,20-23].

An estimated 10-30% of new L1 insertions are full length with the potential for further retrotransposition [11]. Negative evolutionary selection leads to depletion of the full-length elements and there are only about 5000 full-length elements in the human genome [2]. Most of these are old and mutated, and current estimates of the number of potentially active full length L1s stand at about 80–100 per individual [11,14]. Next generation sequencing (NGS) studies have predominantly focused on detecting L1 insertional mutagenesis through detection of the 3’ end of L1 elements [24-27]. Through these NGS techniques, various groups have identified the insertional burden of L1 in germline and somatic tissues, including various cancers. Few studies, however, focus on the specific detection of full-length L1 elements in these genomes that could potentially be hot for retrotransposition and responsible for the accumulation of further L1 insertion events. This is an important facet of the retrotranspositional burden in cancers because these full length L1 elements have been shown to be capable of extremely high levels of continued retrotransposition in tumors with newly inserted hot L1 copies continuing to be actively mobile [28-30].

Studies that have looked at full-length L1 elements in the reference build of the human genome have indicated that the bulk of retrotransposition occurs from a handful of hot L1 elements [11]. Additionally, full length L1 elements that are polymorphic between individuals are significantly more likely to be hot than fixed, reference full length L1s [11,31]. Recent reports support the idea that different L1 elements are active in different individuals and that single hot L1 elements can result in incredibly high levels of insertional mutagenesis [28,29]. Additionally, hot L1 elements in tumors have recently been shown to mobilize to hundreds of new locations within some tumors, with some of these new insertions also further mobilizing to additional locations [30]. Despite the extensive mutagenesis caused by hot L1s, few attempts have been made to identify full length L1 elements in human populations that may lead to successive insertion events.

In this report, we describe a NGS-based method to identify full length L1 elements in human genomes that we call Sequencing Identification and Mapping of Primed L1 Elements (SIMPLE). Using SIMPLE we have identified 228 polymorphic L1 elements in seven independent individuals. SIMPLE has 94% sensitivity and is robustly able to identify nearly all full length L1 elements in a human genome, making it possible to identify hot L1s capable of causing high degrees of insertional mutagenesis.

## Results

### Description of SIMPLE library generation

Various anchored PCR strategies have been effectively employed to identify mobile element insertions in a whole-genome setting. They all share the use of one primer from within the known sequence of the element, and use different strategies to target a second primer outside of the element. However, there are several significant drawbacks to most of those methods. Those that ligate a linker to a restriction site located randomly outside of the mobile element insertion only create one amplified fragment size from each element. Thus, it is impossible to tell whether duplicate sequences are independent ligations or PCR duplicates. A similar argument can be made for the use of an arbitrary primer to prime outside the mobile element. Furthermore, restriction sites located at different lengths away from the element will amplify with differential efficiencies, as will different arbitrary primers. Thus, these methods require pooling of multiple experiments using different restriction enzymes or primers to come close to saturating the potential insertions. In addition, even those protocols that utilize randomly sheared DNA to apply anchors still use a major ligation step in their preparation. These ligation steps, such as those used in the preparation of Illumina libraries, lead to low levels of chimeric fragment ligation between genomic fragments that can confound data analysis [32].

**Figure 1:**
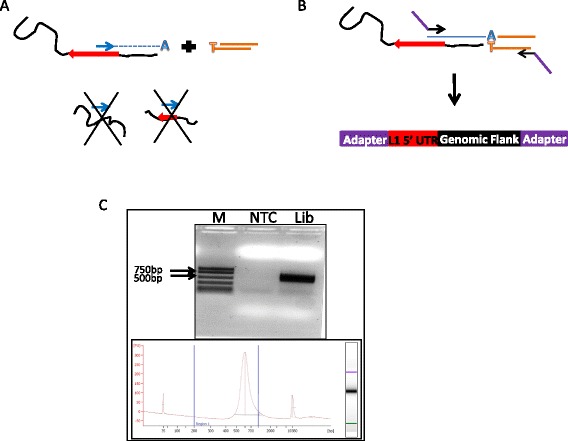
**Description of SIMPLE. (A)** Randomly sheared genomic DNA (black line) is subjected to a single round of primer extension using a primer specific to the L1 5’ UTR (blue arrow). Only DNA fragments containing a full length L1 element (large red arrow) will undergo linear extension from the L1-specific primer. DNA fragments containing no L1 sequence or truncated L1 copies (bottom left and right respectively) will not be extended. Following primer extension, a duplex “t-linker” (orange) is ligated to L1-primer extended ends. **(B)** Following ligation of duplex linkers, adapter sequences needed for Illumina sequencing (purple bars) are added by nested PCR to only those fragments anchored by an L1 extension event. **(C)** Following gel based size fractionation, SIMPLE libraries are run on an agarose gel and analyzed by Agilent BioAnalyzer to confirm size and library quality.

Sequencing Identification and Mapping of Primed L1 Elements (SIMPLE) is a unique L1-detection method based on the principles of random shearing combined with t-linker ligation-mediated PCR that takes advantage of the massive parallelization offered by high throughput NGS technology [33]. A primer specific to the 5’ UTR of full length L1 elements is used in a single round of primer extension in the 5’ direction of the L1 element, which allows for priming on all of the ~5000 full length L1 elements in the human genome and the generation of a single adenine overhang at the end of those extended fragments (Figure 1A). The vast majority of the genome will remain single-stranded so that little besides the extended mobile element fragments will have the A overhang necessary for duplex linker ligation via a 3’ thymidine overhang. Following linker ligation, PCR amplification using L1 5’ UTR and linker-specific primers allows for specific amplification of only those DNA fragments anchored by an L1 extension event (Figure 1B). Additional Illumina adapter sequences are added in a subsequent PCR reaction and the SIMPLE library is size fractionated on an agarose gel before final library amplification (Figure 1C). SIMPLE libraries can be directly loaded onto the next generation sequencing platform for sequence analysis. Thus, each sample is generated from a randomly sheared fragmentation, making it easy to differentiate PCR duplicates from authentic detection of the same element multiple times. In addition, we reasoned that the ligation step used in SIMPLE would be less likely to create chimeras than a traditional linker ligation because of the nature of the 3’ overhangs generated by primer extension and that most of the fragments will remain single-stranded.

### Validation of SIMPLE

**Figure 2:**
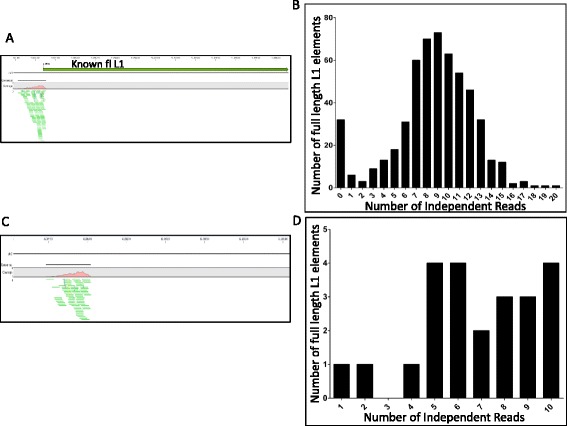
**Validation of 5’-SIMPLE using known full length L1 elements. (A)** CLC Genomics Workbench window view of a representative fixed full length L1 element detected by SIMPLE. L1PA4 represents a known full length L1 element on chromosome 1. Small green lines at bottom are forward SIMPLE reads, reading into the 5’ end of the L1PA4 element. **(B)** Frequency distribution of the number of reference full length L1 elements detected by SIMPLE versus the number of independent SIMPLE hits detected for that element. On average, a reference full length L1 was assayed by nine independent SIMPLE reads, with the vast majority of reference full length L1 elements assayed by at least two independent reads. **(C)** CLC Genomics Workbench window view of a representative non-reference full length L1 element detected by SIMPLE. As in **(A)**, green bars represent forward SIMPLE reads, however in this case, there is no reference full length L1 at the expected site in the genome. **(D)** Frequency distribution of the number of non-reference, known polymorphic full length L1 elements detected by SIMPLE versus the number of independent SIMPLE hits detected for that element. On average, a non-reference full length L1 was assayed by seven independent SIMPLE reads, with the vast majority of reference full length L1 elements assayed by at least two independent reads.

To assess the ability of SIMPLE to detect full length L1 elements from the human genome, we sought to determine the efficiency of SIMPLE at pulling out known, reference full length L1 elements that are fixed in the human population. These L1 elements represent evolutionarily established L1 elements that inserted in a primate genome before the split of *Homo sapiens*, and thus represent L1 elements universally present in all human genomes at a diploid level. We employed SIMPLE to determine how many known, fixed full-length L1 elements on three randomly selected chromosomes we could detect. We limited our initial analysis to three chromosomes to allow a more exhaustive manual analysis of any variants. Of the 543 fixed full-length L1 elements on three randomly selected chromosomes, SIMPLE successfully detected the vast majority with an average read depth of ten independent (having a different linker location) reads per element. Of these, 511/543 (94%) were detected by at least one SIMPLE read, with 502/543 (92%) detected by more than three independent SIMPLE events (reads) (Figure 2A and B). The 32/543 (6%) full-length L1 elements not detected by SIMPLE were located in regions of repetitive DNA (nearly all other genomic L1 elements), which confounds the bioinformatics mapping of these elements.

To determine how SIMPLE performed for non-reference full-length L1 elements (ie, those elements either present as a single copy or not present in any given individual) we assessed the detection rate of previously described polymorphic full-length L1 elements on the same three chromosomes [11,25-27]. While the three chromosomes selected were found to have 152 previously described full-length, polymorphic elements on them, we would only expect a small proportion of these elements to be assayed in our individuals by SIMPLE given the polymorphic nature of these elements. In fact, SIMPLE determined that 23 of these known polymorphic full-length L1 elements were present in our tested population of seven individuals. As with fixed L1 elements, SIMPLE performed well for polymorphic elements, with a median of seven independent SIMPLE reads per polymorphic element (Figure 2C and D). Additionally, the results of our SIMPLE analysis were compared to a lower-throughput method which identified 68 novel full length L1 elements in the genomes of six individuals of diverse backgrounds [31]. Using SIMPLE, we confirmed the presence of 29/68 of these non-reference full length L1 elements in at least one of our seven individuals, including the identification of elements with estimated allele frequencies as low as 2%, many of which were known to be hot for retrotransposition (Additional file 1: Table S3) [31]. These data indicate SIMPLE is a powerful tool to detect virtually all full length L1 elements in a given individual in a single experiment, allowing for the detection of novel non-reference full length L1s that may contribute to mutagenesis by mobilization in these individuals.

### Extent of full length L1 polymorphism

To determine the extent of human full length L1 polymorphism between unrelated individuals, we applied SIMPLE to seven individual genomic DNA samples from non-related Caucasian individuals to assess the degree of full-length L1 polymorphism between these individuals. For ease of analysis, SIMPLE reads mapping to known full-length L1 elements were discarded, and only those reads that did not represent reference elements were taken into account for analysis.

Using SIMPLE, we detected 228 non-reference full-length L1 elements in the genomes of seven unrelated individuals. The range of non-reference, full-length L1 elements varied from a low of 73 per individual to a high of 134 per individual with an average of 96 non-reference full length L1 elements per individual (Table 1). Of the non-reference, full-length L1 elements detected by SIMPLE, 160 were elements present in dbRIP or described by previous studies [24,26,27,31,34]. The remaining 68 non-reference full length L1 elements detected by SIMPLE in these individuals were novel elements that had not previously been described (Table 1, Additional file 2: Table S2).

**Table 1:** **Summary of polymorphic full length L1 elements in the seven individuals tested in this study**

**Individual**	**Total polymorphics**	**Total Unique (New)**
**1**	73	7 (6)
**2**	75	8 (8)
**3**	134	28 (14)
**4**	120	13 (6)
**5**	96	6 (3)
**6**	91	4 (2)
**7**	80	3 (2)
**Avg.**	***96***	***10 (6)***

Column two represents the total number of polymorphic full length elements detected in a particular individual. Column three represents the number of non-reference full length L1 elements unique to one individual (and the number of those that represent novel elements).

**Figure 3:**
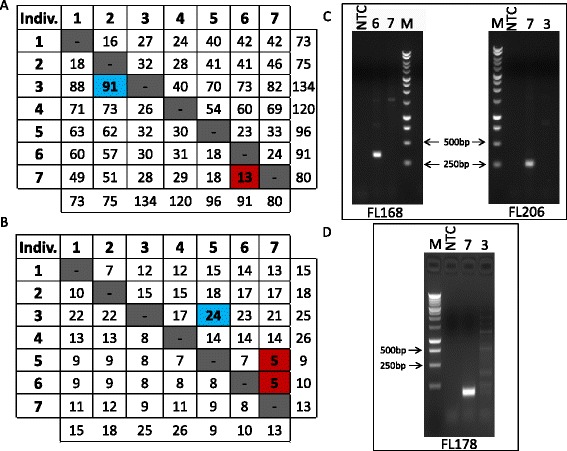
**Significant variation in the number of non-reference full length L1 elements is observed between individuals. (A)** Two-way matrix showing the total number of polymorphic full length L1 loci present in one individual but not in another. For example, the first row represents the number of polymorphic full length L1 elements present in individual one but not in individuals two through seven (left to right). Total numbers of polymorphic full length L1 elements per individual are included at the end of each row and the bottom of each column for reference. Numbers vary from a low of 13 elements in individual seven that are not found in individual six (red square) to a high of 91 elements that are found in individual three, but are not found in individual two (blue square). **(B)** Similar to **(A)** except showing the subset of only novel (previously unreported) polymorphic full length L1 loci present in one individual but not in another. For example, the first row represents the number of novel polymorphic full length L1 elements present in individual one but not in individuals two through seven (left to right). Total numbers of novel polymorphic full length L1 elements per individual are included at the end of each row and the bottom of each column for reference. Numbers vary from a low of five elements in individuals five and six that are not found in individual seven (red squares) to a high of 24 elements that are found in individual three, but are not found in individual five (blue square). **(C)** Representative gels depicting 5’ flank PCR of two randomly selected non-reference full length L1 elements. Lane labels: NTC=no template control, M=1 kb DNA ladder, Arabic numerals=individual number as per Table 1. **(D)** Representative gel depicting 3’ flank PCR of non-reference full length L1 element. Lane labels are as in **(C)**.

**Figure 4:**
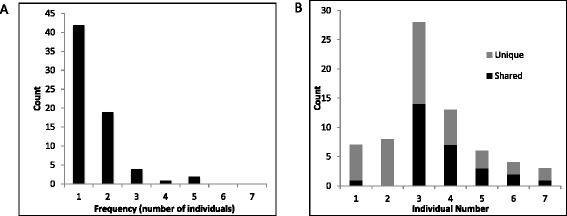
**Further SIMPLE validation. (A)** Frequency distribution showing the number of novel (ie, previously undescribed) non-reference SIMPLE-detected full length L1 elements (Y-axis) versus the number of individuals in which they were detected (X-axis). A majority (42/68, 62%) of novel non-reference full length L1 elements were detected in only one individual, however 26/68 (38%) were detected in at least two individuals and 7/68 (10%) were detected in three or more individuals. **(B)** Graph showing the number of novel non-reference full length L1 elements found in each individual. Numbers ranged from three novel non-reference elements in individual seven to 28 in individual three. The average number of novel non-reference full length L1 elements per individual was ten.

To get a better understanding of the frequency distribution of non-reference, full-length L1 elements detected by SIMPLE, we performed pair-wise comparisons of the SIMPLE-detected non-reference L1 elements between each of the 7 individuals (Figure 3). Our data reveal any two individuals shared between 29 and 94 non-reference full length L1 elements with the average number of non-reference L1s shared between any two individuals being 52 (Additional file 3: Figure S1A). Similar pairwise comparisons revealed any one individual has between 13 and 91 non-reference, full-length L1 elements that are not present in another single individual with an average of 44 non-reference, full-length L1 elements not shared between any two individuals (Figure 3A). Analysis of novel non-reference, full-length L1 elements (ie, those not previously reported) revealed individuals contained between 9 and 26 novel non-reference full length L1 elements, with an average of 15 novel non-reference elements per individuals (Figure 4B). Of these, most are unique to a single individual, but as many as 10% are shared between at least two individuals in our sample (Figure 3B, Figure 4A, Additional file 3: Figure S1A). Taken together, these data indicate an individual genome may contain upwards of 90 non-reference full length L1 elements with the potential to be active.

### Validation of SIMPLE-detected polymorphisms

Non-reference, full-length L1 polymorphisms detected by SIMPLE were subjected to PCR validation to confirm the presence of the non-reference elements. We randomly chose 15 non-reference, full-length L1 elements detected by SIMPLE to perform PCR based validation using L1 junction PCR to amplify across the 5’ L1 junction. This method allowed us to determine the 5’ junction of the non-reference L1 element by Sanger sequencing of PCR product. We successfully validated 15/15 (100%) SIMPLE-detected L1 polymorphisms by 5’ junction PCR and confirmed the insertions by Sanger sequencing of the 5’ junction (Figure 3C, Additional file 4: Table S4). Additionally, a random sample of validated 5’-SIMPLE reads was also validated for 3’ junctions. We successfully validated seven full length SIMPLE junctions at the 3’ end, and were able to determine polyA tail length, L1 EN cleavage site, and target site duplication size for these polymorphic elements, which showed these elements displayed the normal features expected of insertion by retrotransposition (short target site duplications, polyA tails, and L1 endonuclease consensus cleavage sites) (Additional file 5: Table S5). Taken together, these data show SIMPLE is a robust method for detecting non-reference full length L1 elements.

### Distribution of non-reference full length L1 elements

We next analyzed the chromosomal distribution of non-reference, full-length L1 elements detected in our study. SIMPLE successfully detected non-reference L1 elements on all 22 autosomes and X with no significant bias for any chromosome to harbor either a particularly high or particularly low number of non-reference, full-length L1 elements. Novel full-length L1 elements were detected on all chromosomes except 8 and 19 (Additional file 6: Figure S2). Analysis of the number of individuals in our cohort sharing any given non-reference full length L1 reveals that the majority of novel full-length L1 elements detected by SIMPLE are present in only a single individual, indicating they occur at low allele frequency. However, surprisingly, a sizeable number (38%) of novel full length L1s were detected in at least two independent individuals. In fact, 7/68 (10%) novel full length L1 elements detected by SIMPLE were present in at least three independent individuals (Figure 4A). Additionally, our data show that any individual contains on average 10 unique non-reference, full-length L1 elements with more than half of those (on average, six) having not been previously described (Figure 4B and Table 1). However, the vast majority of the novel full length L1s detected by our study represent relatively rare alleles that are limited to only one or two individuals in our sample. To highlight this, we compared the allele frequencies of the 68 novel full length L1s described by this study to the 160 previously described polymorphic full length L1s also detected in our seven individuals. We find that while the previously described polymorphic L1 elements range from rare to common among our seven individuals, the majority of the novel polymorphic full length L1 elements detected by SIMPLE are unique to a single individual (Additional file 7: Figure S3). Taken together, these data suggest that the extent of low frequency (rare) non-reference, full-length L1 insertion polymorphisms in the population may be greater than expected by previous estimates [11,12,35].

### Allele frequency estimates of novel full length L1s

**Figure 5:**
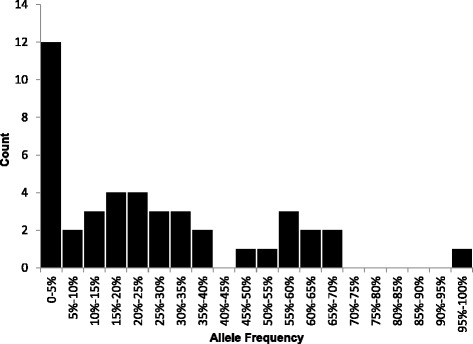
**Allele frequency determination of full length polymorphic L1s.** The allele frequency of 40 randomly selected full length polymorphic L1s detected by SIMPLE was determined by screening a panel of 80 unrelated individuals from four distinct populations. Most polymorphic L1s tested were found to be low allele frequency elements, with only very few elements existing at allele frequencies above 50%.

Because our study identified numerous novel full-length L1s that we anticipate are present at low allele frequency based on their frequency within our seven-individual population (Additional file 7: Figure S3), we wanted to determine the allele frequencies of the polymorphic full length L1 elements detected in our study in a larger population of individuals. We conducted a population study to determine the allele frequency of 43 randomly chosen polymorphic elements in 80 individuals from four geographically diverse backgrounds (African American, Asian, German Caucasian, and South American). The vast majority (79%) of full-length polymorphic L1 elements tested existed at an allele frequency less than 50%, with most (58%) existing at less than 25% allele frequency. Additionally, 33% of polymorphic full length L1s were rare in the population, with allele frequencies less than 10% (Figure 5). These frequencies remained consistent whether we looked at the total population, or within each of the four subpopulations (Additional file 8: Figure S4). Together, these data suggest that the polymorphic elements detected by SIMPLE represent low frequency polymorphisms in the population that are younger and most likely to be active.

### Application of SIMPLE to L1 3’ ends

**Figure 6:**
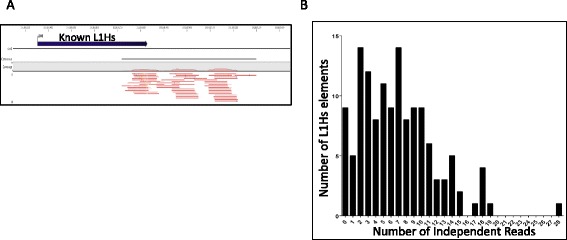
**Adaptation of SIMPLE to detect L1 3’ ends. (A)** CLC Genomics Workbench window view of a representative fixed human specific L1 element (L1Hs) detected by 3’-SIMPLE. L1Hs represents a known human specific L1 element on chromosome 1. Small red lines at bottom are reverse 3’-SIMPLE reads, reading into the3 end of the L1Hs element. **(B)** Frequency distribution of the number of reference L1Hs elements detected by 3’-SIMPLE versus the number of independent 3’-SIMPLE hits detected for that element. On average, a reference L1Hs element was assayed by seven independent 3’-SIMPLE reads, with the vast majority of reference L1Hs elements assayed by at least two independent reads.

To determine if SIMPLE could be easily adapted to detect all novel L1 insertions, we sought to modify SIMPLE to target the 3’ end of human specific L1 elements (L1Hs) because the vast majority of new L1 insertions are 5’ truncated [2,20,36,37]. We designed primers targeting only the youngest, most active L1 subfamilies and performed SIMPLE targeting L1Hs 3’ ends in a single individual. To determine the sensitivity of L1 SIMPLE for L1 3’ ends, we performed a similar analysis to that done in Figure 2B. By mapping 3’-SIMPLE data to the human genome reference build and assaying three random chromosomes for detection of known L1Hs 3’ ends, we determined SIMPLE performs similarly for L1 3’ ends as it does for 5’ ends, detecting 93% of known L1Hs elements on the three chromosomes assayed (Figure 6A). Like with 5’-SIMPLE, those elements not detected by 3’-SIMPLE are elements located in areas rich in repetitive DNA sequences, compounding mapping. Like 5’-SIMPLE, 3’-SIMPLE is able to detect L1Hs 3’ ends with high confidence with an average of seven independent 3’-SIMPLE hits per known L1Hs (Figure 6B). These results support the use of SIMPLE for detection of *de novo* L1 insertion events, which would prove useful in studying L1 mutagenesis in various cancer types.

## Discussion

Full length polymorphic L1 elements are responsible for virtually all of the disease-causing retroelement insertions and the bulk of retroelement mobilization observed in tumors. We have developed a robust tool to identify full-length polymorphic L1 elements in individual genomes, which also has potential to be applied to the detection of *de novo* somatic L1 insertion events in tumors or other samples. Because DNA fragmentation for SIMPLE occurs by random shearing of genomic DNA by sonication, SIMPLE is able to detect L1 elements that may be located too distally from restriction enzyme cut sites used by other high-throughput sequencing methods [25,26]. Additionally, SIMPLE has benefits over other fosmid-based approaches used in the past in that it is extremely high throughput, allowing the detection of essentially all genomic L1 elements in a single SIMPLE reaction [31]. Those rare elements missed by SIMPLE, which reside within repetitive regions of the genome (ie, other L1 elements) are likely a function of read length. It is possible that future iterations of SIMPLE using longer reads would allow for exact mapping of reads that lie partially within repetitive genomic regions.

Using SIMPLE, we have determined the typical individual contains about 100 non-reference, full-length L1 elements. Based on previous estimates, we expect 63/100 of these elements to be active, with 54/100 exhibiting particularly high levels of activity [31]. These 100 non-reference, full-length L1 elements are in addition to the active polymorphic L1 elements present in the human genome reference build, indicating the potential burden of active L1s in any one individual exceeds previous estimates of 80–100 elements per individual [11]. Additionally, our results indicate that the number of non-reference, full-length L1 elements in the population likely exceeds previous estimates given the finding that a number of novel elements detected by SIMPLE were present in multiple individuals. Further, the variation we see between individuals in terms of the number of non-reference, full-length L1 elements implies there are likely individual differences in each person’s “retrotransposition potential”, with some individuals in a population being more prone to L1 jumping than others, which is consistent with previous hypotheses [38] as well as with recent data showing some hot L1s are differentially active between different cancer [28-30].

It is interesting to note that although all seven individuals assayed in this study were Caucasian, there is still significant variation between individuals with respect to non-reference, full-length L1 elements. We anticipate, however, that analysis of diverse populations will yield similar results in terms of absolute numbers to those reported here. We would expect an individual from any single population to harbor the average 100 non-reference full length L1 elements with the only difference being in the identity of the particular elements found in that individual. Two individuals from similar backgrounds will share a greater number of non-reference elements than two individuals from diverse backgrounds, but both individuals will have ~100 non-reference full length L1 elements each regardless of their background.

## Conclusion

SIMPLE represents a useful tool not only for both population and tumor-based studies of L1 insertion polymorphism. The vast majority of L1 retrotransposition is likely to occur due to the approximately 100 polymorphic L1 loci in each individual. Work looking at L1 mobilization in tumors has identified a number of tumor types that appear to support L1 mobilization (colorectal, prostate, lung, ovarian) as well as others that do not (glioblastoma, multiple myeloma) [25-27,39,40]. While this preference for particular tumor types can be explained by cell-specific factors affecting L1 mobilization, there is also significant variation in the extent of L1 mobilization within cancers of the same type that could be due to differences in polymorphic, active L1 elements between two individuals [27,40]. Using SIMPLE, it may be possible to determine the nature of biases in L1 retrotransposition rates between different individuals or tumors and attribute them to differences in the number or location of active L1 elements between two individuals. Additionally, with the application of SIMPLE to L1Hs 3’ ends, it can be used to determine the total levels of L1 retrotransposition in different individuals and tumors.

## Methods

### Cell lines and oligonucleotides

Fibroblast cell lines GM01631, GM01632, GM05510, GM05568, GM16094, GM15983, and GM08207 were obtained from the Coriell Institute (Camden, NJ). Cell lines were maintained in EMEM supplemented with non-essential amino acids, sodium pyruvate, and 10% fetal bovine serum. DNA oligonucleotides and duplex linkers were obtained from Integrated DNA Technologies (Coralville, IA). Oligonucleotide sequences used in this study are presented in Additional file 9: Table S1.

### 5’ SIMPLE library generation

Genomic DNA from fibroblast cell lines was extracted using the DNEasy Blood and Tissue Kit from Qiagen (Germantown, MD). DNA was sheared to approximate 750-1200 bp using a Diagenode BioRuptor on High, 30s on/30s off for 12 minutes. 50 ng of sheared gDNA was subject to a primer extension reaction using Taq polymerase and a L1 5’ UTR specific primer (L15’UTRP1) which sits ~100 bp from the start of the L1 element. Phosphorylated duplex T-linkers (IDT, Coralville, IA) were ligated using T4 DNA ligase. First round PCR was performed for 20 cycles using primers L15’UTRP1 and LinkerP1. One-million fold dilution of PCR I was performed and 1uL of this dilution was subjected to a nested PCR using primers L15’UTRP2 and LinkerP1 for 25 cycles. PCR products were run on a gel and a gel slice at ~500-700 bp was extracted using the Qiagen Gel Extraction kit. Following extraction, the final library was amplified using Phusion polymerase (Thermo, Waltham, MA) for 12 cycles as per the Illumina library generation protocol, and gel purified to yield the final 500-700 bp library.

### Library quantitation and illumina sequencing

Illumina sequencing libraries were quantified by qPCR and checked for quality by Agilent BioAnalyzer trace at Elim Biopharmaceuticals (Hayward, CA). 100 bp paired end Illumina sequencing was performed on an Illumina HiSeq 2000 by Elim Biopharmaceuticals (Hayward, CA).

### Reference genome masking

Reference repetitive element annotation files were downloaded from UCSC genome browser as GTF files and used to annotate the GRCh37 (hg19) reference genome in CLC Genomics Workbench (CLC Bio, Cambridge MA). Two custom GFF annotation files were also generated: 1) representing known polymorphic elements and 2) representing all known full length L1 elements plus 600 bp of upstream genomic flanking sequence.

To identify and annotate 5’ L1 regions of the genome, the first 300 base pairs of the L1.3 consensus sequence was aligned to the human reference genome (CRCh37/hg19) via NCBI BLAST (blastn algorithm). Initial search parameters were relaxed to increase sensitivity for older, more mutated elements (word size=7, gap cost=3, gap extension cost=3, match score=2, mismatch penalty=−3, minimum E score of 10). Blast output was generated in tabular format. Because many of the hits obtained from this search consisted of isolated smaller regions (20-30 bp) that were independent of any identifiable L1 element, we further filtered for those hits where alignments were made across >=250 bp of the 300 bp query. This greatly improved our specificity while maintaining sensitivity to older, more mutated elements. Tabular format blast results were converted to GFF via Perl scripting, and an additional 600 bp of flanking sequence was added upstream and downstream to each matching position.

Annotation of 3’ L1 ends and flanking sequenced was performed using a similar strategy as above, except that initial identification of 3’ end locations was based on the UCSC Genome Browser hg19 RepeatMasker table track, which allowed for subfamily classification based on all available sequence from the insertion.

### 5’ SIMPLE mapping strategy

FASTQ sequencing files were analyzed for quality using FastQC. PCR duplicate reads were removed using custom in-house Perl scripting. Sequence read mappings were performed using CLC Genomics Workbench. Single end genomic flank reads were mapped using a two-pronged mapping strategy. First, reads that either mapped to within 600 bp of known, fixed full length L1 elements or those reads that did not map unambiguously were discarded, as they represented either known elements or unmappable reads. The remaining reads were then mapped uniquely to an annotated GRCh73/hg19 reference genome to identify novel polymorphic or potentially *de novo* full length L1 elements.

### Polymorphic full length L1 allele frequency determination

#### DNA samples

Confirmed novel L1 elements were genotyped to determine the allele frequency of the insert on a DNA panel of 80 diverse individuals (20 African Americans, 20 Asians, 20 Europeans and 20 South Americans) obtained from the Coriell Institute for Medical Research, Camden, NJ. The efficacy of each primer pair was initially assessed through PCR using human DNA cell line HeLa (ATCC CCL-2) and/or chimpanzee DNA cell line Clint (S006006), confirming the size of the predicted PCR products.

#### PCR primer design

BLAT was used to locate candidate L1 sequences and flanking DNA these sequences in the hg 19 human reference genome, and add 1,000 bp of flanking sequence both upstream and downstream. Sequences were then screened through RepeatMasker confirming the absence of candidate L1 inserts from the hg 19 sequence and localizing unique genomic regions for primer design. For the most part, prospective PCR primers flanking each candidate L1 locus were designed using Primer3 software. Once screened through the BLAT genome browser, primers were selected for PCR if they were predicted to amplify a single locus. In addition, a virtual PCR was performed for each locus using the in silico function of BLAT to compute the expected PCR product size, annealing temperature, and further verify that only one locus would be amplified. For candidate L1 loci directly flanked by substantial stretches (more than 1 kb) of repetitive genomic sequence, primers were designed manually. In such instances, flanking sequences (up to 1 kb) were screened using the BLAT genome browser and aligned with closely reported matches (usually 4–12 loci) in BioEdit (Ibis Biosciences). Point mutations, insertions, and/or deletions specific to the candidate L1 loci were manually positioned toward the 3’ end of the primers. In addition, internal primers, purposed to anneal to the 3’ end of a full length human specific L1 element, were available [41].

#### PCR analysis

PCR assays were performed in four stages. (1) An empty site PCR analysis was initially conducted using HeLa DNA. External primers flanking each candidate L1 locus were used to amplify PCR products matching the predicted empty site band lengths previously generated using BLAT’s in silico PCR feature. This assay was capable of amplifying PCR products no larger than 1,500 bp. As a result, alleles lacking the L1 insertion were exclusively amplified. Neither alleles possessing an L1 insertion nor unsuitable primer pairs amplified PCR products. Thus it was impossible to distinguish suitable primers pairs flanking L1 inserts that were homozygous present in HeLa DNA from an unsuccessful reaction (failure to amplify an existing empty site). Therefore, given the human specific nature of the predicted L1 inserts, chimpanzee DNA was used in a control PCR analysis examining each primer pair that failed to amplify an empty site during the initial PCR. (2) An internal primer test was conducted to verify the presence of novel L1 inserts and verify that they were full length. These PCR analyses were performed using DNA samples from the individual(s) in which the inserts were originally identified, an external primer flanking the predicted 3’ end of the L1, and a 3’ internal primer [41]. (3) A long amplification PCR was conducted to further confirm the presence or absence of candidate L1 insertion characterized in the former stages. This assay was capable of amplifying PCR products up to 15 kb. Thus the flanking external primers were used to amplify the entire L1 insertion. (4) An allele frequency PCR analysis was conducted using a DNA panel of 80 individuals (20 African Americans, 20 Asians, 20 Europeans and 20 South Americans). This assay subjected each sample to both an empty site assay (stage 1) and an internal primer test (stage 2).

Stages 1, 2, and 4 were performed in 25 μL reactions containing 25 ng of template DNA, 200 nM of each oligonucleotide primer, 1.5 mM MgCl2, 10× PCR buffer (50 mM KCl, 10 mM TrisHCl; pH 8.4), 0.2 mM deoxyribonucleotide triphosphates and 1 to 2 U Taq DNA polymerase. PCR reactions were performed as follows: initial denaturation at 94°C for 60 seconds, followed by 32 cycles of denaturation at 94°C for 30 seconds, 30 seconds at primer annealing temperature (determined previously with HeLa DNA), and extension at 72°C for 30 seconds. PCR reactions were terminated with a final extension at 72°C for 2 minutes. Fractionation of 20 μL of each PCR product was performed in a horizontal gel chamber on a 2% agarose gel containing 0.2 μg/mL ethidium bromide for 45 minutes at 200 V. UV-fluorescence was used to visualize the amplified DNA fragments.

Stage 3 was performed using Takara LA-Taq (long amplification) DNA polymerase (Clontech Laboratories, Inc., Mountain View, CA). These PCR reactions were performed in 25 μL reactions containing 25 ng of template DNA and LA-Taq PCR reagents according to the manufacturer’s suggested protocol: initial denaturation at 94°C for 1 minute and 20 seconds, followed by 32 cycles of denaturation at 94°C for 20 seconds, 20 seconds at the optimized annealing temperature, and a long extension step at 68°C for eight minutes and 30 seconds. These long-amplification reactions were terminated with a final extension at 68°C for 10 minutes. PCR products were size fractionated on a 1% agarose gel for 150 minutes to 180 minutes at 150 V. UV-fluorescence was used to visualize the DNA fragments.

### 3’-SIMPLE library generation

Library generation for 3’-SIMPLE was performed in the same manner as 5’-SIMPLE with the following modifications. Primer extension used L1HsP1 primer. First round PCR was 22 cycles rather than 20 and used primers L1HsP1 and LinkerP1. Second round PCR used primers L1HsP2 and LinkerP1. All remaining steps of 3’-SIMPLE library generation were identical to those for 5’-SIMPLE.

### 3’-SIMPLE mapping strategy

Raw reads were processed for quality and duplicate removal in the same manner as for 5’-SIMPLE. A similar two-pronged mapping strategy was used for 3’-SIMPLE involving mapping reads to known L1Hs elements and then taking unmapped reads and mapping unambiguously to an annotated GRCh37/hg19 genome.

## Additional files

Additional file 1: Table S3. Detection of previously reported polymorphic full length L1s. This table shows details for 68 previously identified polymorphic L1s [31], some of which were also detected by this study. Additional file 2: Table S2. Polymorphic full length L1 elements identified in this study. This table shows details for all 228 polymorphic full length L1s identified by this study. Additional file 3: Figure S1. Pairwise comparison of polymorphic L1 elements shared between any two individuals. (A) Two-way matrix showing the total number of polymorphic full length L1 loci shared between any two individuals. For example, the first row represents the number of polymorphic full length L1 elements shared between individual one and each of individuals two through seven (left to right). Total numbers of polymorphic full length L1 elements per individual are included at the end of each row and the bottom of each column for reference. Numbers vary from a low of 29 elements shared between individuals two and seven (red square) to a high of 94 elements shared between individuals three and four (blue square). (B) Similar to (A) except showing the subset of only novel (previously unreported) polymorphic full length L1 loci shared between any two individuals. For example, the first row represents the number of novel polymorphic full length L1 elements shared between individual one and each of individuals two through seven (left to right). Total numbers of novel polymorphic full length L1 elements per individual are included at the end of each row and the bottom of each column for reference. Numbers vary from a low of zero elements shared between individuals one and five and individuals two and five (red squares) to a high of eight elements that are shared between individuals one and two and individuals three and four (blue squares). Additional file 4: Table S4. Validation of SIMPLE-detected full length L1s by 5’ flank PCR. This table shows details of the validation of SIMPLE-detected polymorphic L1s using 5’ junction PCR. Additional file 5: Table S5. Validation of SIMPLE-detected full length L1s by 3’ flank PCR. This table shows details of the validation of SIMPLE-detected polymorphic L1s using 3’ junction PCR. Additional file 6: Figure S2. Chromosomal distribution of polymorphic full length L1 elements across seven individuals. Polymorphic full length L1 elements were detected by SIMPLE on all autosomes and chromosome X without any apparent bias for one genomic region over another. Novel full length L1 elements (grey) were also detected across nearly all autosomes and chromosome X without apparent bias. Additional file 7: Figure S3. Allele frequency of known and novel full length L1s among the seven individuals in our population. Graph shows the frequency (as a percentage) of known (blue bars) or novel to this study (red bars) polymorphic full length L1 elements within our population. Previously described polymorphic L1s show an even distribution with some representing rare alleles (present in only one or two individuals) and others representing common alleles (present in all or most individuals). However, the novel polymorphic L1s show a distribution much more skewed to rare alleles, with most novel polymorphic L1s being unique to only a single individual. Additional file 8: Figure S4. Allele frequency of 40 selected polymorphic full length L1s from four diverse populations. Histogram showing the allele frequencies of 40 randomly selected polymorphic full length L1s broken down by population subtype. 20 unrelated individuals from each of four geographically distinct ethnic groups (African American, Asian, German Caucasian, and South American) were tested for the presence or absence of each selected polymorphic full length L1. Additional file 9: Table S1. List of oligonucleotides used in this study. NNNNN represent random barcodes used for multiplexing.
