# Supplementary figures and images for: Sequencing, identification and mapping of primed L1 elements (SIMPLE) reveals significant variation in full length L1 elements between individuals
Source: BMC Genomics. 2015 Mar 21;16(1):220. doi: 10.1186/s12864-015-1374-y (PMC4381410; doi:10.1186/s12864-015-1374-y)

## Slide 1
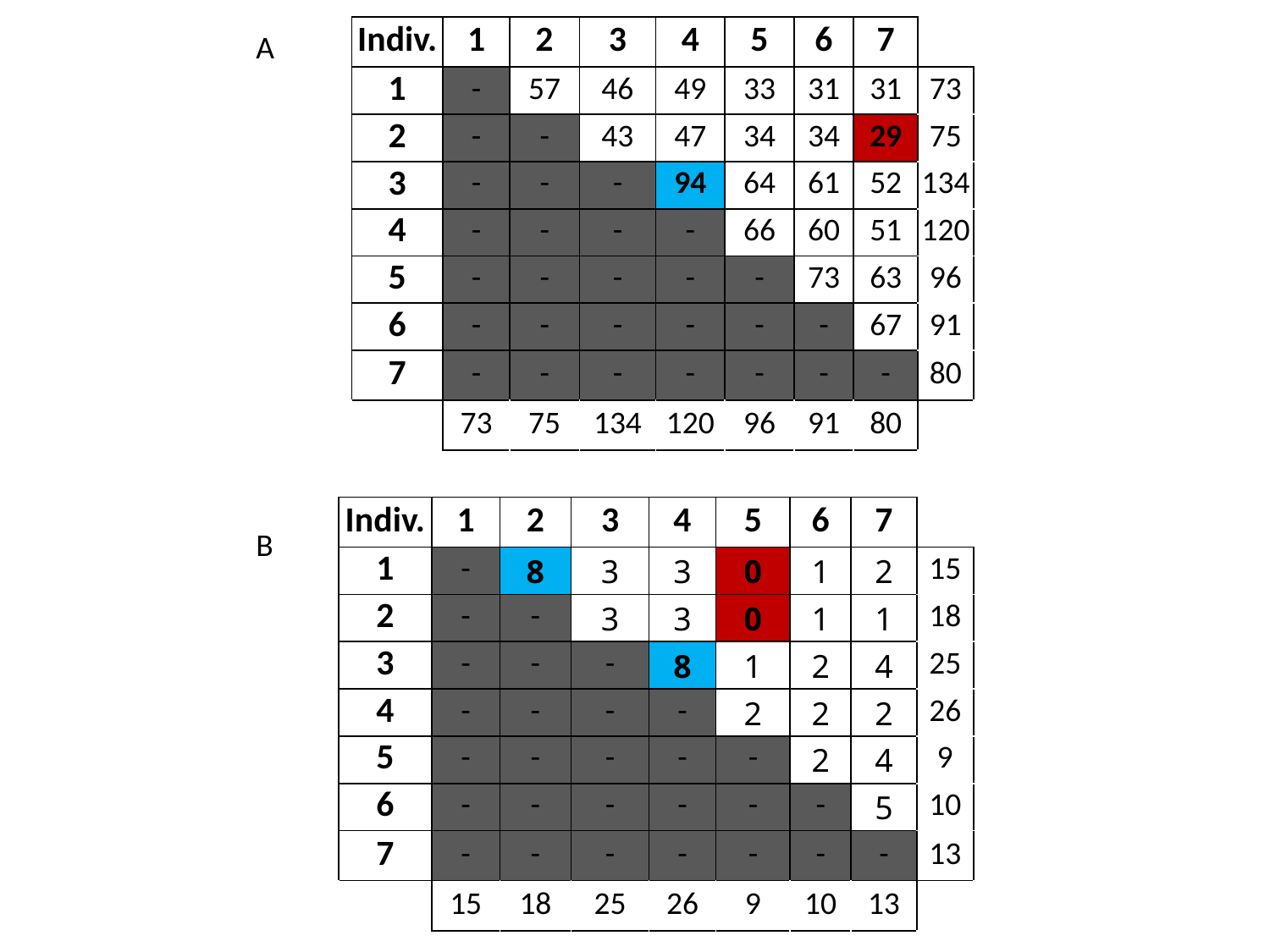

| Indiv. | 1 | 2 | 3 | 4 | 5 | 6 | 7 | |
| --- | --- | --- | --- | --- | --- | --- | --- | --- |
| 1 | - | 57 | 46 | 49 | 33 | 31 | 31 | 73 |
| 2 | - | - | 43 | 47 | 34 | 34 | 29 | 75 |
| 3 | - | - | - | 94 | 64 | 61 | 52 | 134 |
| 4 | - | - | - | - | 66 | 60 | 51 | 120 |
| 5 | - | - | - | - | - | 73 | 63 | 96 |
| 6 | - | - | - | - | - | - | 67 | 91 |
| 7 | - | - | - | - | - | - | - | 80 |
| | 73 | 75 | 134 | 120 | 96 | 91 | 80 | |
A
| Indiv. | 1 | 2 | 3 | 4 | 5 | 6 | 7 | |
| --- | --- | --- | --- | --- | --- | --- | --- | --- |
| 1 | - | 8 | 3 | 3 | 0 | 1 | 2 | 15 |
| 2 | - | - | 3 | 3 | 0 | 1 | 1 | 18 |
| 3 | - | - | - | 8 | 1 | 2 | 4 | 25 |
| 4 | - | - | - | - | 2 | 2 | 2 | 26 |
| 5 | - | - | - | - | - | 2 | 4 | 9 |
| 6 | - | - | - | - | - | - | 5 | 10 |
| 7 | - | - | - | - | - | - | - | 13 |
| | 15 | 18 | 25 | 26 | 9 | 10 | 13 | |
B

Supplement: Additional file 3: Figure S1. — Pairwise comparison of polymorphic L1 elements shared between any two individuals. (A) Two-way matrix showing the total number of polymorphic full length L1 loci shared between any two individuals. For example, the first row represents the number of polymorphic full length L1 elements shared between individual one and each of individuals two through seven (left to right). Total numbers of polymorphic full length L1 elements per individual are included at the end of each row and the bottom of each column for reference. Numbers vary from a low of 29 elements shared between individuals two and seven (red square) to a high of 94 elements shared between individuals three and four (blue square). (B) Similar to (A) except showing the subset of only novel (previously unreported) polymorphic full length L1 loci shared between any two individuals. For example, the first row represents the number of novel polymorphic full length L1 elements shared between individual one and each of individuals two through seven (left to right). Total numbers of novel polymorphic full length L1 elements per individual are included at the end of each row and the bottom of each column for reference. Numbers vary from a low of zero elements shared between individuals one and five and individuals two and five (red squares) to a high of eight elements that are shared between individuals one and two and individuals three and four (blue squares). [file 12864_2015_1374_MOESM3_ESM.pptx]

## Slide 1
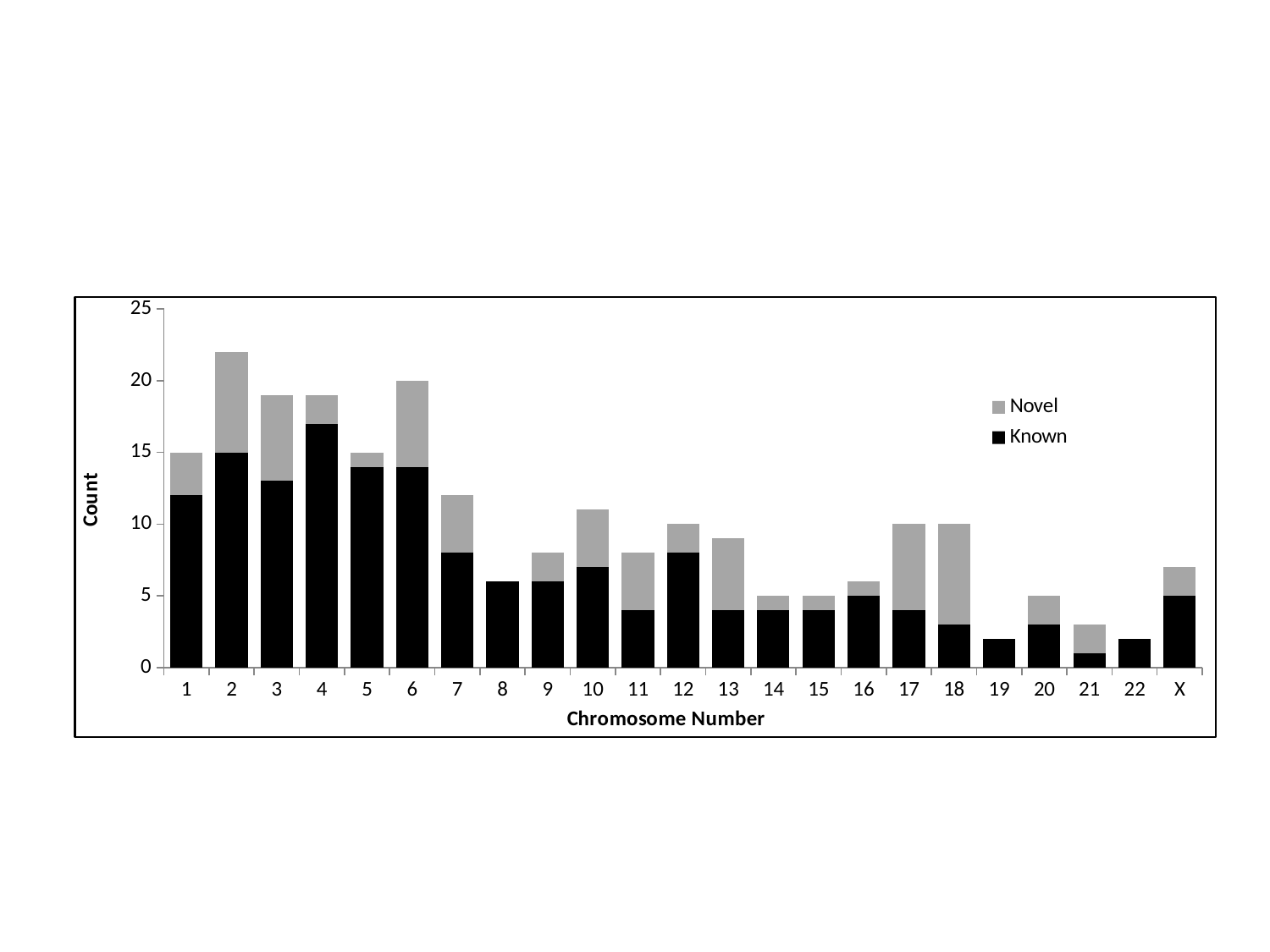

### Chart
| Category | | |
|---|---|---|
| 1 | 12.0 | 3.0 |
| 2 | 15.0 | 7.0 |
| 3 | 13.0 | 6.0 |
| 4 | 17.0 | 2.0 |
| 5 | 14.0 | 1.0 |
| 6 | 14.0 | 6.0 |
| 7 | 8.0 | 4.0 |
| 8 | 6.0 | 0.0 |
| 9 | 6.0 | 2.0 |
| 10 | 7.0 | 4.0 |
| 11 | 4.0 | 4.0 |
| 12 | 8.0 | 2.0 |
| 13 | 4.0 | 5.0 |
| 14 | 4.0 | 1.0 |
| 15 | 4.0 | 1.0 |
| 16 | 5.0 | 1.0 |
| 17 | 4.0 | 6.0 |
| 18 | 3.0 | 7.0 |
| 19 | 2.0 | 0.0 |
| 20 | 3.0 | 2.0 |
| 21 | 1.0 | 2.0 |
| 22 | 2.0 | 0.0 |
| X | 5.0 | 2.0 |

Supplement: Additional file 6: Figure S2. — Chromosomal distribution of polymorphic full length L1 elements across seven individuals. Polymorphic full length L1 elements were detected by SIMPLE on all autosomes and chromosome X without any apparent bias for one genomic region over another. Novel full length L1 elements (grey) were also detected across nearly all autosomes and chromosome X without apparent bias. [file 12864_2015_1374_MOESM6_ESM.pptx]

## Slide 1
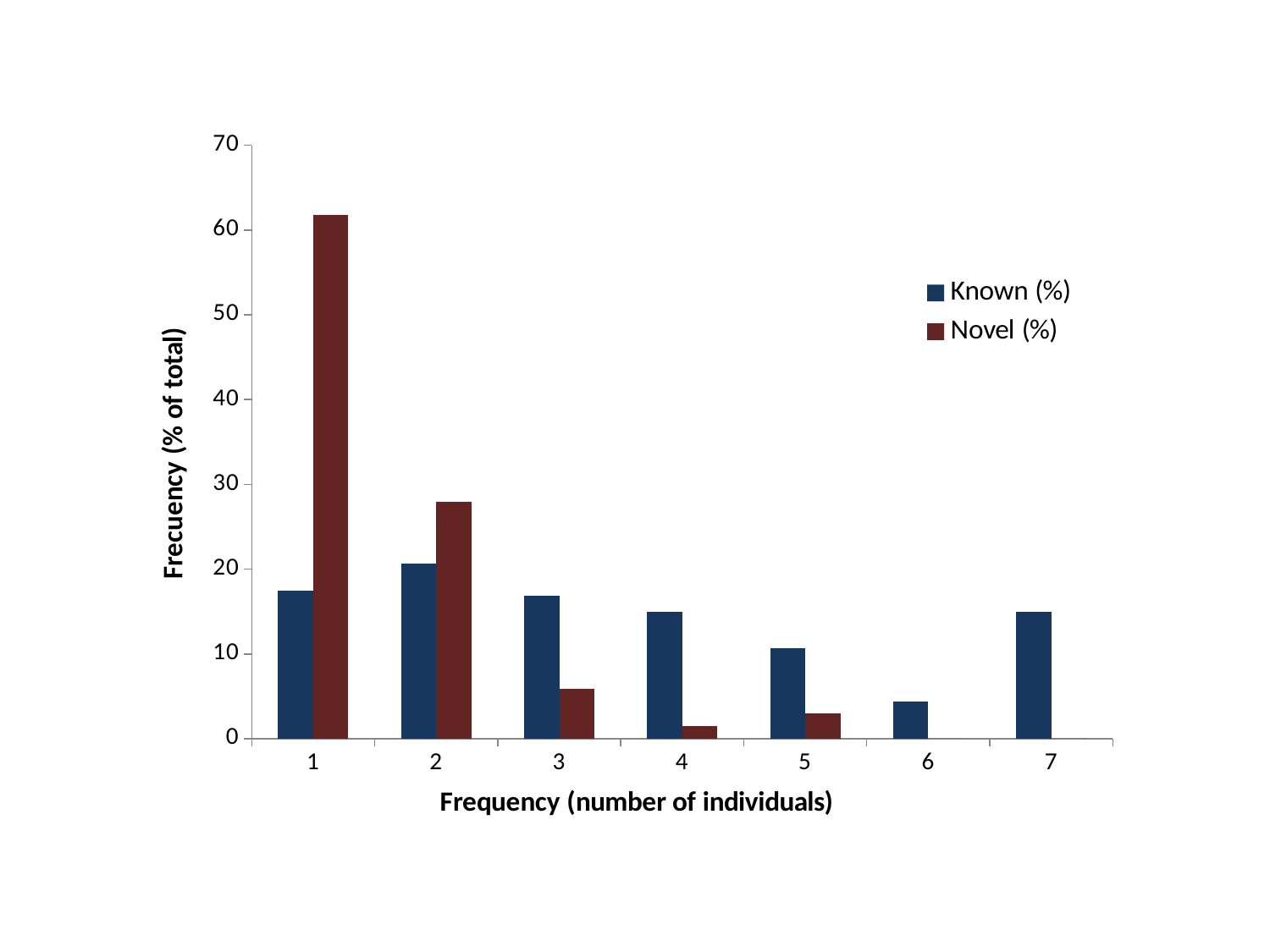

### Chart
| Category | Known (%) | Novel (%) |
|---|---|---|
| 1 | 17.5 | 61.76470588235294 |
| 2 | 20.625 | 27.941176470588236 |
| 3 | 16.875 | 5.88235294117647 |
| 4 | 15.0 | 1.4705882352941175 |
| 5 | 10.625 | 2.941176470588235 |
| 6 | 4.375 | 0.0 |
| 7 | 15.0 | 0.0 |

Supplement: Additional file 7: Figure S3. — Allele frequency of known and novel full length L1s among the seven individuals in our population. Graph shows the frequency (as a percentage) of known (blue bars) or novel to this study (red bars) polymorphic full length L1 elements within our population. Previously described polymorphic L1s show an even distribution with some representing rare alleles (present in only one or two individuals) and others representing common alleles (present in all or most individuals). However, the novel polymorphic L1s show a distribution much more skewed to rare alleles, with most novel polymorphic L1s being unique to only a single individual. [file 12864_2015_1374_MOESM7_ESM.pptx]

## Slide 1
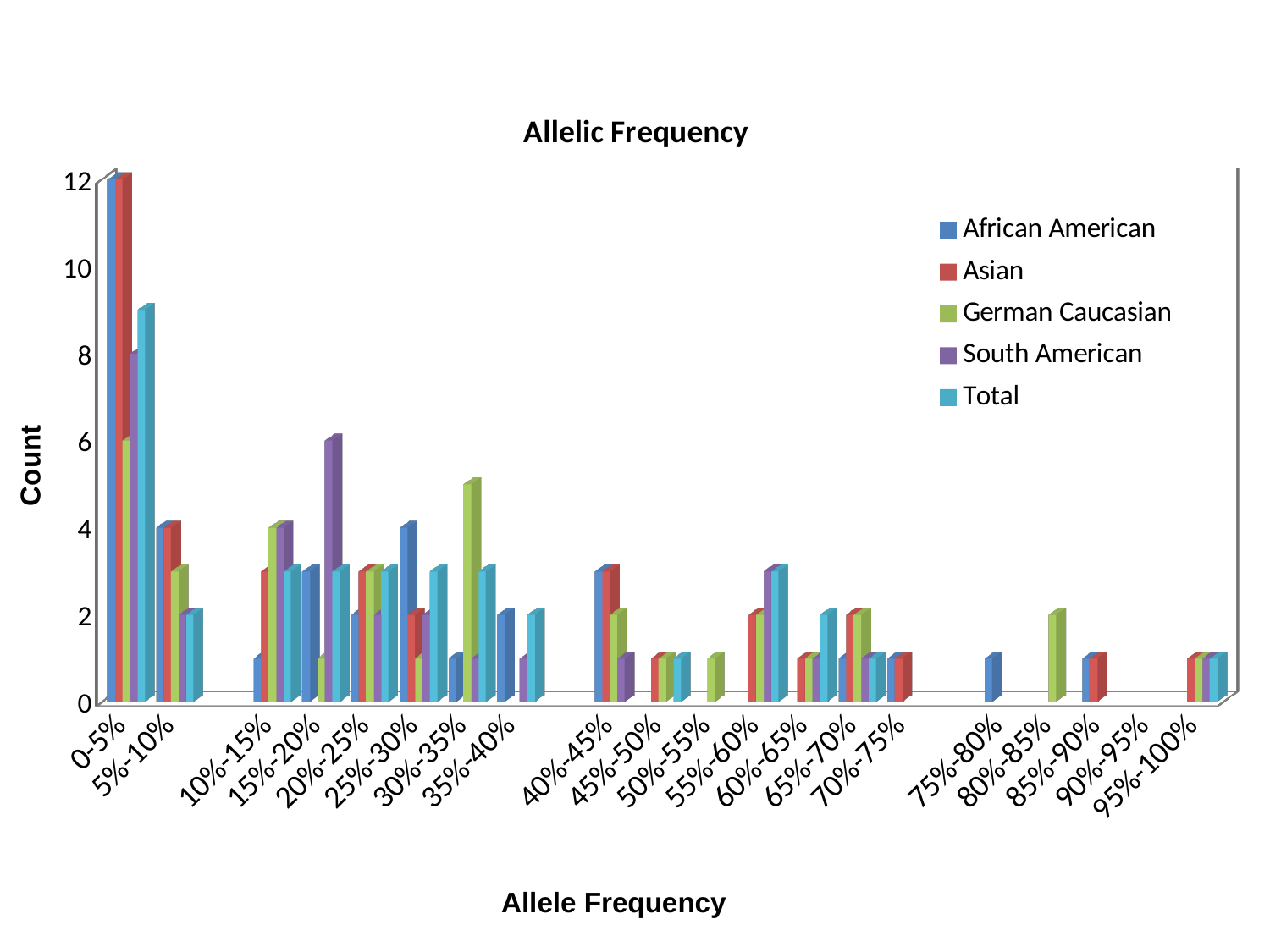

[unsupported chart]
Count
Allele Frequency

Supplement: Additional file 8: Figure S4. — Allele frequency of 40 selected polymorphic full length L1s from four diverse populations. Histogram showing the allele frequencies of 40 randomly selected polymorphic full length L1s broken down by population subtype. 20 unrelated individuals from each of four geographically distinct ethnic groups (African American, Asian, German Caucasian, and South American) were tested for the presence or absence of each selected polymorphic full length L1. [file 12864_2015_1374_MOESM8_ESM.pptx]
