# Supplementary material for: Sequencing, identification and mapping of primed L1 elements (SIMPLE) reveals significant variation in full length L1 elements between individuals
Source: BMC Genomics. 2015 Mar 21;16(1):220. doi: 10.1186/s12864-015-1374-y (PMC4381410; doi:10.1186/s12864-015-1374-y)
Supplement: Additional file 9: Table S1. — List of oligonucleotides used in this study. NNNNN represent random barcodes used for multiplexing. [file 12864_2015_1374_MOESM9_ESM.docx]

| **Primer Name** | **Primer Sequence** |
| --- | --- |
| L15’UTRP1 | GGTGAGGCAATGCCTCGCCCTGCTT |
| L15’UTRP2 | CTCGGCATTCCTGCTGAACCGCTCTTCCGATCTNNNNN*CCACTGTCTGGCACTCCCTAGTGAGATGAA* |
| LinkerP1 | ACACTCTTTCCCTACACGACGCTCTTCCGATCT |
| T-linker top | 5’/Phos/GATCGGAAGAGCGTCGTGTAGGGAAAGAGTGTAGA |
| T-linker bottom | TCTACACTCTTTCCCTACACGACGCTCTTCCGATCT |
| L1HsP1 | ATAGCATTGGGAGATATACCTA |
| L1HsP2 | CTCGGCATTCCTGCTGAACCGCTCTTCCGATCTNNNNNATACCTAATGCTAGATGACACA |
